# Identifying pathophysiological bases of disease in COVID-19

**DOI:** 10.1186/s41231-020-00067-w

**Published:** 2020-09-22

**Authors:** Carla J. Goldin, Ramiro Vázquez, Fernando P. Polack, Damian Alvarez-Paggi

**Affiliations:** 1 INFANT Foundation, Buenos Aires, Argentina; 2 CONICET, Buenos Aires, Argentina; 3 Early Drug Development Group (E2DG), Boulogne-Billancourt, France; 4 Fondazione Istituto Italiano di Tecnologia, Milan, Italy

**Keywords:** SARS-CoV-2, COVID-19, Pathophysiology, RAAS, Risk factors, Comorbidities, Endotypes

## Abstract

COVID-19 is an infectious disease caused by the SARS-CoV-2 virus that can affect lung physiology encompassing a wide spectrum of severities, ranging from asymptomatic and mild symptoms to severe and fatal cases; the latter including massive neutrophil infiltration, stroke and multiple organ failure. Despite many recents findings, a clear mechanistic description underlying symptomatology is lacking.

In this article, we thoroughly review the available data involving risk factors, age, gender, comorbidities, symptoms of disease, cellular and molecular mechanisms and the details behind host/pathogen interaction that hints at the existence of different pathophysiological mechanisms of disease. There is clear evidence that, by targeting the angiotensin-converting enzyme II (ACE2) –its natural receptor–, SARS-CoV-2 would mainly affect the renin-angiotensin-aldosterone system (RAAS), whose imbalance triggers diverse symptomatology-associated pathological processes. Downstream actors of the RAAS cascade are identified, and their interaction with risk factors and comorbidities are presented, rationalizing why a specific subgroup of individuals that present already lower ACE2 levels is particularly more susceptible to severe forms of disease. Finally, the notion of endotype discovery in the context of COVID-19 is introduced.

We hypothesize that COVID-19, and its associated spectrum of severities, is an umbrella term covering different pathophysiological mechanisms (endotypes). This approach should dramatically accelerate our understanding and treatment of disease(s), enabling further discovery of pathophysiological mechanisms and leading to the identification of specific groups of patients that may benefit from personalized treatments.

## Introduction

The recently described SARS-CoV-2 virus is the latest addition into the group of pathogenic human coronaviruses (HCoV). The *Coronavirinae* subfamily encompasses four different genera: *alpha, beta, gamma* and *deltacoronavirus*. The genetic and serologic groups *alfa*- and *betacoronavirus* includes pathogens that mainly infect mammals (except pigs) [1]. The normally circulating 229E and NL63 are *alphacoronaviruses* whereas OC43 and HKU1 are *betacoronaviruses*. During the last twenty years, three additional HCoVs from zoonotic origin have surfaced: SARS-CoV, MERS-CoV and SARS-CoV-2,all belonging to the *betacoronavirus* genus. While the usual HCoV are normally associated with common cold symptoms, these last pathogens may elicit infections that range from asymptomatic carrier to severe pneumonia, leading to acute respiratory distress syndrome (ARDS). A common feature of SARS-CoV and SARS-CoV-2 is that viral attachment occurs via interaction of the viral spike (***S***) protein —which is primed by the Transmembrane Serine Protease 2 (TMPRSS2)— to the host angiotensin-converting enzyme 2 (ACE2), allowing viral entry [2, 3]. Interestingly, this feature is shared with the NL63 HCoV, while the other HCoVs employ different receptors such as dipeptidyl peptidase 4 and aminopeptidase N [4]. The ***S***/ACE2 interaction gives place to a cross-talk point between viral infection and the renin-angiotensin-aldosterone system (RAAS), and there is mounting evidence that this interplay may crucially affect disease severity (see below).

SARS-CoV-2 causes COVID-19, a disease that presents a wide range of clinical manifestations, from asymptomatic to severe ARDS and may result fatal due to respiratory insufficiency, stroke, thrombotic complications [5] and, finally, multi organic failure [6]. Although an accurate mechanistic description is lacking, it is proposed that an uncontrolled and excessive release of pro-inflammatory cytokines (called “cytokine storm”) may cause some of the symptoms, including shock and tissue damage, and massive neutrophil infiltration [7]. Current consensus is that older people, immunocompromised or patients with significant underlying conditions and comorbidities such as diabetes and hypertension are more likely to experience severe COVID-19 symptoms [8].

Assessment of the mechanisms underlying SARS-CoV-2-induced disease and severity has focused mainly on the immunopathological features [7, 9, 10], and have resulted in some unexpected findings: the unusual seroconversion processes involving IgM and IgG titers among infected patients [11], the age-dependent cytokine storm-induced reduction and functional exhaustion of CD4^+^ and CD8^+^ T cells —both critical to eliminate virus-infected cells and for achieving successful recovery [12]—, and the possible link between severity and genetic variations in chemokine receptors and blood group loci [13], among others. These findings clearly hint at the existence of distinct pathophysiological bases of disease in COVID-19. In addition, other actors have been identified or proposed, such as endocrine and metabolic pathways [14] and the role of infected endothelial cells [15] in disease severity. However, a comprehensive and cohesive evaluation of these factors is lacking. In the following sections, we present a detailed review attempting to identify molecular bases of disease severity based on the specifics of host/pathogen interplay, with an emphasis on the endocrine-immune interactions involved. Finally, we speculate that COVID-19 is actually an umbrella term that includes several pathophysiological mechanisms, known as endotypes, originated in the individual-specific host/pathogen interactions, which simultaneously depend on the functional status of the RAAS.

## The entry point of SARS-CoV-2: ACE2 and TMPRSS2

### ACE2 is a central component of the RAAS

**Fig. 1:**
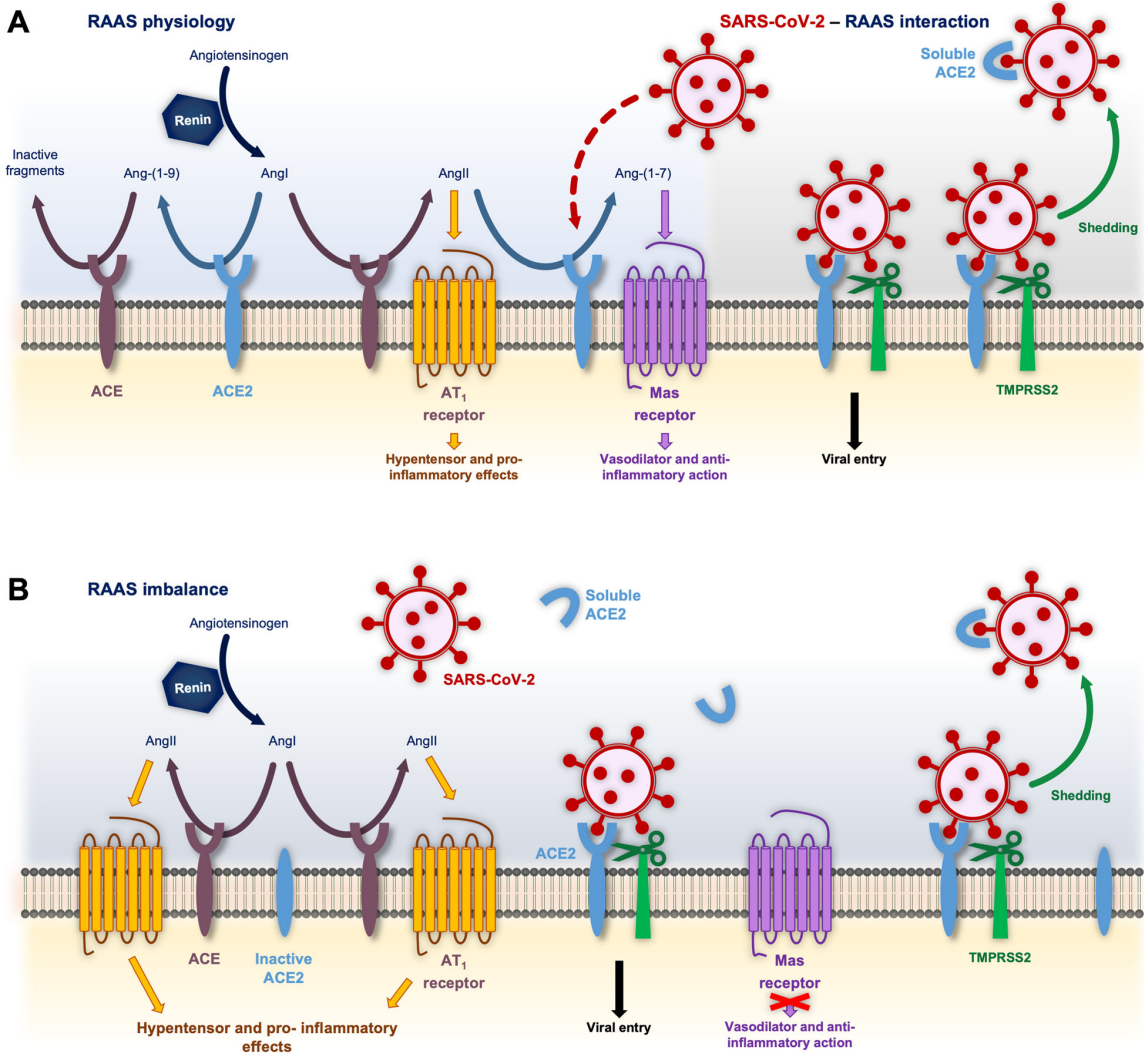
**a** Key pivotal modulating and antagonistic roles of ACE and ACE2 in the RAAS, and SARS-Cov-2 binding to ACE2 and TMPRSS2. ACE catalyzes the conversion of AngI into AngII, thereby inducing hypertensive and pro-inflammatory effects, while ACE2 mediates the formation of angiotensin-(1–9) from AngI. ACE2 also counters ACE activity by reducing AngII bioavailability and increasing Ang-(1–7) formation, which acts as a vasodilator and exerts antiinflammatory activities through Mas receptors. SARS-CoV-2 interacts and downregulates ACE-2. **b** In this context, an imbalance in ACE2/Ang-(1–7) and ACE/AngII axes would be critical in the development of severe COVID-19 symptomatology

The coronaviruses SARS-CoV, SARS-CoV-2 and NL63-CoV rely on binding of their ***S*** protein to ACE2 [2, 16] for attachment and cell entry, being able to infect many of the organs where it is expressed [17–19]. Human ACE2 is a transmembrane enzyme that contains different functional domains: a *C*-terminal anchoring region, a *N*-terminal signal peptide region, and an extracellular HEXXH zinc-binding metalloprotease domain [20–22]. ACE2 is a member of the RAAS, that involves a variety of hormones and enzymatic reactions whose primary role consists of regulating the homeostasis of the cardiovascular and renal systems [23, 24], playing also a critical function in inflammatory response [25]. This system consists of two main axes: the classic angiotensin-converting enzyme (ACE)-angiotensin II-AT1 receptor, and the ACE2-angiotensin-(1–7)-Mas receptor axis, that was discovered rather recently (Fig. 1).

Both ACE and ACE2 are found in the cytoplasmic membrane of arterial and venous endothelial cells, and arterial smooth muscle cells [26, 27]. ACE2 is expressed in several organs such as the heart, kidney, lung and testes, among others [17, 19]. In particular, it is present in human nasal epithelium, alveolar and small intestinal cells [28]. ACE and ACE2 have been largely studied as pivotal members of the RAAS. As shown in Fig. 1, they play antagonistic roles by processing the renin-cleaved decapeptide angiotensin both competitively or in an alternate fashion. The main role of ACE2 is countering ACE activity by reducing angiotensin 2 (AngII) —a potent vasopressor and sodium-and-water retaining octapeptide— bioavailability and increasing angiotensin-(1–7) (Ang-(1–7)) formation —a vasodilator and diuretic peptide—, although alternative catalytic pathways exist [29–31]. In this context, an imbalance in ACE2/Ang-(1–7) and ACE/AngII axes may be critical in the development of cardiovascular diseases [32]. Activation of the ACE-mediated classic axis leads to deleterious effects: vasoconstriction, fibrosis, migration, fluid retention, thrombosis and inflammation; on the other hand, the ACE2-centered via exerts protective vasodilation, and antithrombotic, antiarrhythmic and anti-inflammatory actions [33, 34].

### *S* induces downregulation of ACE2 after complex formation

The extracellular domain of ACE2 can be cleaved from the transmembrane domain by at least two different enzymes, ADAM metallopeptidase domain 17(ADAM17) and TMPRSS2, and the resulting soluble protein is released into the bloodstream and ultimately excreted into urine [3, 35]. TMPRSS2 is a type II transmembrane serine protease expressed in the airway epithelial cells and several tissues. It participates not only in SARS-CoV-2 infection, but is also required by other respiratory viruses such as human influenza and metapneumoviruses [36, 37]. TMPRSS2 increases the infective capacity of both NL63 ***S***- and SARS CoV ***S***- pseudotyped HIV as well as authentic SARS-CoV and SARS-CoV-2, even in cells with low levels of ACE2 expression, inducing ACE2 shedding and thereby loss of its physiological function [2, 37–39] (Fig. 1). The role of TMPRSS2 enabling viral entry would consist of: i) ACE2 cleavage, promoting viral uptake, and ii) ***S*** cleavage in two distinct sites, allowing viral fusion to a host membrane [3, 37, 40]. In the case of SARS-CoV, both mechanisms are independent since ACE2 processing by TMPRSS2 is necessary to increase SARS-CoV ***S***-driven entry but is dispensable for SARS-CoV ***S*** activation [3]. In addition, SARS-CoV ***S*** and, to a lesser extent, NL63-CoV ***S*** can also induce ADAM-17 dependent cleavage of ACE2 in vitro [38, 39].

The interaction energies of different CoV ***S*** proteins with ACE2 have been shown to follow a NL63-CoV < < SARS-CoV < SARS-CoV-2 [41, 42] order due to overlapping but not identical binding interfaces and amino acid variations in the ***S*** protein among the different viruses [42, 43]. Interestingly, although the interaction energy between SARS-CoV-2 receptor binding domain (RBD) and ACE2 is higher than that observed for SARS-CoV RBD, SARS-CoV-2 RBD is less accessible, resulting in similar apparent binding affinities [44]. The interplay between ***S*** and ACE2 complex formation and the activation of host proteases suggests that although the viral entry mechanisms are similar between NL63-CoV, SARS-CoV and SARS-CoV-2, ACE2 downregulation levels might correlate with the binding affinities involved in complex formation, which may play a key part in COVID-19 symptomatology.

## SARS-CoV-2-induced RAAS imbalance results in inflammation and other severe COVID-19 symptoms

The role of unbalanced RAAS as a central player in ARDS and acute lung injury is nowadays well established [45]. ACE-generated AngII triggers inflammatory processes, stimulating proliferation of mononuclear cells and regulating the recruitment of proinflammatory cells (by expressing vascular permeability factors and adhesion molecules, among others) [46], rendering the AngII-degrading ACE2 as an essential actor for homeostasis. ACE2-deficient animals are significantly more susceptible to severe pulmonary damage in the context of SARS coronavirus, Influenza H7N9 virus or bacteria infections, as well as LPS inhalation [47–50]. These facts hint at a counterintuitive role of ACE2 expression levels in determining the severity of SARS-CoV-2 infection: although SARS-CoV-2 entry is dependent on ACE2, it is established that lower levels of this molecule can cause exacerbated inflammation, at least to some extent. In mice, during lung infection the initial reduction of pulmonary ACE2 is crucial for recruiting the inflammatory neutrophils to combat the infection, and the subsequent recovery of pulmonary ACE2 is critical to prohibit exuberant neutrophil accumulation. It was found that ACE2 modulated neutrophil infiltration through IL-17-mediated STAT3 signaling, which also recruits factors from the inflamed microenvironment [48]. Confounding factors that either prevent the ACE2 dynamics from occurring or disrupt it are detrimental to the host, resulting in either compromised host defense capability or heightened inflammatory lung diseases [48]. Evidence shows that SARS-CoV ***S*** protein, which is not infective, exerts proinflammatory effects: intraperitoneally inoculation with recombinant SARS-CoV ***S*** worsens the severity of acid aspiration-induced acute lung injury in wild-type mice [47], increasing AngII levels in the lungs. Furthermore, when AngII receptor type 1 (AT1R) was blocked, acute lung injury in ***S***-treated mice was attenuated [47]. Complement system also plays a role: infection of C3 deficient mice with mouse-adapted SARS-CoV exhibited less respiratory dysfunction and fewer neutrophils, inflammatory monocytes and lower cytokine levels in lungs than wild-type mice [51].

Endothelial cells continuously express ACE2, constituting an optimal infection target for SARS-CoV and SARS-CoV-2 [52, 53]. This allows infection spreading and affects the RAAS ecosystem of each organ, and entails direct injury in the endothelium leading to endotheliitis [53], higher vascular permeability and hemostatic dysfunction [54]. In addition, such constitutive expression would explain the significant thrombotic disorders recently reported in the autopsies of COVID-19 patients [55]. In addition, many of the observed severe symptoms or causes of death are represented over different organs. The major complications observed are ARDS [56–60], acute cardiac injury [56, 58, 60], heart failure [56, 61], shock [56, 58, 60], acute kidney injury [56, 58–60], hypoxic encephalopathy [56], lymphopenia [60] and acute pulmonary embolism [62], which could all be at least partially ascribed to disbalancing of the RAAS.

Recent studies have shown a high incidence of neurological symptoms in COVID-19 cases. Although most of them are minor (like headache, nausea, and a loss of sense of smell and taste), more complicated symptomatology, such as convulsions, stroke and thrombotic complications have been also reported [63–65]. There is a strong possibility that these complications arise, at least in part, from downregulation of ACE2. It is now heavily documented that one of the important effects of ACE2 /Ang-(1–7)/mas receptor axis is on the brain and cerebral blood vessels [66], exerting protection against stroke [67] and there is evidence supporting the overall concept that the aging increases the sucseptivility of the cerebrovasculature to the effects of RAAS disbalance [68].

**Fig. 2:**
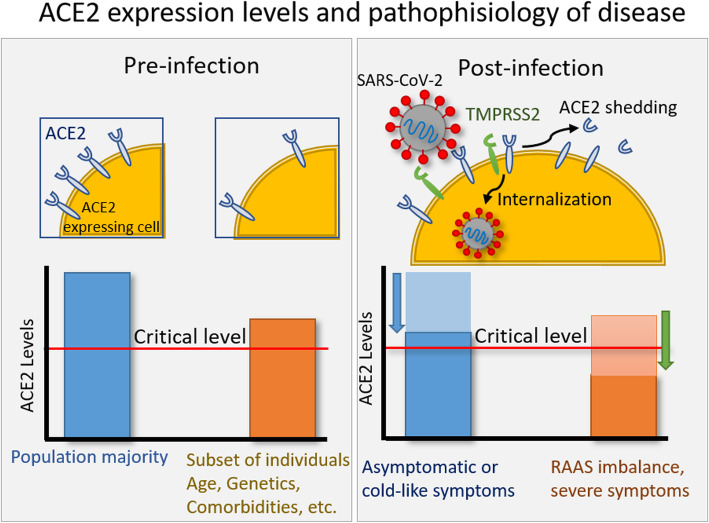
The effect of ACE2 expression levels on COVID-19 disease and severity. Age, genetics, and different comorbidities affect the pre-infection ACE2 expression levels in a subset of individuals (left), rendering them susceptible to severe forms of disease. During infection (right), upon interaction of SARS-CoV-2 S protein with ACE2 and TMPRSS2, ACE2 levels are downregulated. Those individuals with low pre-infection ACE2 levels reach a threshold critical value corresponding to the onset of severe symptomathologies due to RAAS imbalance

Taking into account the results obtained in mice models and SARS-CoV, and its similarities with SARS-CoV-2, there is strong evidence that differences in expression levels of ACE2 in the context of SARS-CoV-2 infection may constitute a molecular basis of exacerbated inflammation (Fig. 2). This is further supported by the observation that patients with severe COVID-19 show an increase in neutrophil count and in the neutrophil-to lymphocyte ratio and elevated levels of proinflammatory cytokines [7], consistent with in vivo results of neutrophil infiltration after ACE2 downregulation [48]. Moreover, a correlation between the ratio of pro- and anti-inflammatory cytokine concentrations and symptom severity has been observed [69]. It can be speculated that only a few cases of HCoV-NL63-induced severe cases have been reported due to the lower ***S***/ACE2 complex affinity that results in milder dysregulation of ACE2 levels. However, patients with a subgenotype of HCoV-NL63 were hospitalized with severe lower tract infection in 2018. That subgenotype presented one mutation in its RBD that enhances viral entry into host cells, hinting at ACE2 downregulation underlying the severe symptomatology [70]. Furthermore, another few cases of HCoV-NL63-positive patients (82 yo median age) emerged, showing distress syndrome, with symptoms including pneumonia, multiple organ failure and death, although the subgenotype is unknown [71, 72].

Crucially, the RAAS presents a complex interplay with cyclooxygenase-2 (COX-2 [73, 74]) which is rapidly inducible in several cell types in response to growth factors, cytokines, and pro-inflammatory molecules. It is largely responsible for the onset of inflammation, participating in the synthesis of proinflammatory prostaglandins and triggers production of other proinflammatory chemokines and cytokines, and playing a role in hypertension [75]. Interestingly, while inhibition of COX-2 expression exerts a suppressive effect on lung inflammation [76], it has been shown that both ***S*** and the nucleoprotein (***N***) of SARS-CoV upregulate COX-2 [77] through different molecular mechanisms. Considering the high identity sequence of ***S*** and ***N*** proteins between both viruses (75 and 90%, respectively [78]), SARS-CoV-2 may also elicit upregulation of COX-2, further exacerbating inflammation.

Finally, ACE genotypes may affect the SARS-CoV-2/RAAS interplay. A critical ACE polymorphism consists of the presence (insertion, I) or absence (deletion, D) of a 287-bp *Alu* sequence in intron 16 [79], being the D allele associated with increased activity [80]. Intensive unit care patients bearing the D allele or DD genotype are more susceptible not only to develop ARDS, but also to present a less favourable outcome [81], with a higher risk of mechanical ventilation [82–84]. Interestingly, the D allele was in a higher frequency in those patients who developed the most severe symptoms of SARS-CoV infection [82]. In addition, a recent analysis of the prevalence of ACE (I/D) genotype in different countries showed that as the I/D allele frequency ratio increases, the COVID-19 recovery rate in each country also increases [85].

## Pathophysiological contributions of COVID-19 risk factors

### Hypertension and diabetes

Despite the large number of SARS-CoV-2 positive patients, understanding COVID-19 pathogenesis remains elusive. Available reports indicate that the most frequent comorbidity in severe COVID-19 is hypertension, followed by diabetes and coronary heart disease [86]. Reports on the clinical characteristics of patients with COVID-19 show that 2.5 to 14.5% of SARS-CoV-2 positive patients present cardiovascular diseases, 12,8 to 56.6% of patients present hypertension and 5.3 to 33.8% patients have diabetes [87].

Ang-(1–7) has multiple beneficial cardiovascular effects: protection against heart failure, natriuretic and antithrombotic, among others [88]. In a mice model of ang II-dependent hypertension, blood pressures were higher in the ACE2-deficient mice than in wild-type specimens [89]. ACE2 expression in heart is also necessary for structural and functional regulation. After a myocardial infarction, ACE2-deficient mice presented an enhanced susceptibility to a second event, with increased mortality, infarct expansion and adverse ventricular remodeling. Loss of ACE2 also led to increased neutrophil infiltration in the infarct and peri infarct regions, resulting in upregulation of inflammatory cytokines [90].

The kidney is highly sensitive to RAAS perturbation. Several studies demonstrated an increased activity of this system involved in the development and progression of diabetic renal damage [91]. In mice models of either type 1 and type 2 diabetes mellitus, ACE2 expression is elevated in early stages of diabetic nephropathy while decreasing in the late phase of the disease, suggesting that ACE2 may participate in a compensatory mechanism in the diabetic kidney prior to illness onset [92]. Moreover, in a murine model of diabetic nephropathy, recombinant ACE2 administration improves kidney function and structure [93]. In agreement with these results, it was shown that ACE2 expression is decreased in the tubules in human diabetic nephropathy [94]. The imbalance of the RAAS system in favor of AngII in the context of diabetes results in a more severe kidney damage in males than in females, which is even increased if ACE2 is downregulated [95, 96].

### Age

Age is a major factor affecting the severity of COVID-19 disease, correlating with both susceptibility to infection and manifestation of clinical symptoms. Therefore, incidence of clinical cases in countries with younger populations is expected to be lower than older population countries, despite the prevalence of other comorbidities [97]. It has been proposed that AT1R-mediated signaling is involved in the aging process per se by promoting several age-related pathologies, such as cardiovascular diseases, diabetes, chronic kidney failure, dementia, osteoporosis and even cancer [98, 99]. Increased AngII bioavailability due to reduced catabolism may result in overactivation of these receptors. In line with this, several authors have observed that ACE2 expression levels are reduced with age [26, 100, 101].

**Fig. 3:**
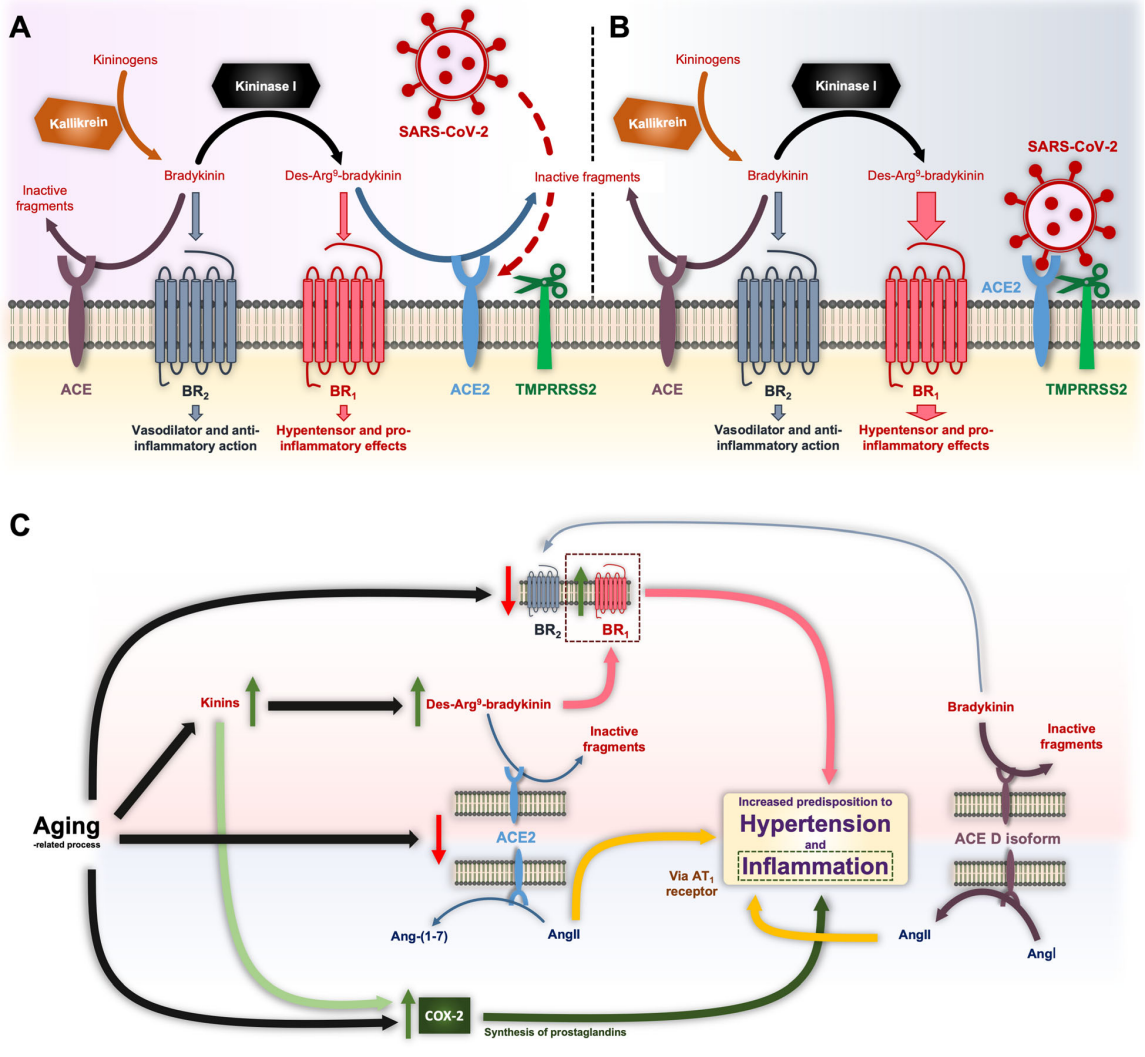
**a** Action of ACE and ACE2 enzymes in the KKK system. ACE degrades bradykinin, a vasodilator peptide acting mainly through BR2 receptors. This kinin can be also converted by kininase I into des-Arg9-bradykinin, which promotes vasoconstriction and pro-inflammatory effects upon interaction with BR1 receptors. ACE2 participates in the degradation of des-Arg9-bradykinin, a process eventually inhibited by SARS-CoV-2-induced ACE2 downregulation. **b** SARS-CoV-2-mediated imbalance in the KKK system with predominant pro-inflammatory effect of des-Arg9-bradykinin. **c** Age-related variations in the RAAS, KKK system and COX-2 and ACE D isoform as enhancers of the susceptibility of older adults to present severe COVID-19 symptoms

ACE and ACE2 exert catalytic effects on several proteins beyond the RAAS. This apparent promiscuity confers these enzymes enough plasticity to reach the same physiological effects through alternative pathways, thereby producing quicker, more intense and coordinated responses. Thus, age-related alteration in the ACE/ACE2 activity does not only affect the physiology of the RAAS, but also another particular system in which both proteins have a prominent role: the kininogen-kinin-kallikrein (KKK). As shown in Fig. 3a**,** ACE has been demonstrated to be one of the primary proteases responsible for the hydrolysis of the kinin bradykinin and, to a lesser extent, its derivative des-Arg^9^-bradykinin. It is worth remarking that ACE is considered first a kininase, being known as kininase II [102], and then an angiotensinase, due to its »80-fold higher affinity for bradykinin with respect to AngI (4). In fact, the cough presented by some patients treated with ACE inhibitors has been attributed to the blockade of the bradykinin metabolism [103]. ACE2, on the other hand, degrades des-Arg^9^-bradykinin but no other forms of bradykinin (4). There are two types of kinin receptors: BR1, selectively sensitive to kinins lacking the C-terminal Arg residue like des-Arg^9^-bradykinin; and BR2, optimally stimulated by the full sequence of bradykinin. While BR2 is constitutive and widely expressed in different tissues and mediates vasodilator and anti-inflammatory effects, the gene encoding BR1 is regulated by a promoter region with binding sites for transcription factors such as the activator protein-1 and the nuclear factor kappa B (NFkB), which are up-regulated during inflammation [104]. By acting on BR1 receptors, des-Arg^9^-bradykinin induces vasocontraction and pro-inflammatory actions [104]. Thus, SARS-CoV-2 infection would favor the overactivation of the BR1 with deleterious effects in the affected tissue (Fig. 3b). In agreement with this, recent works point out to des-Arg^9^-bradykinin as a key mediator of lung injury caused by LPS [104, 105]. By employing ACE2-deficient mice, Sodhi and collaborators found that this enzyme is crucial in counteracting such mechanism giving its ability to inactivate des-Arg^9^-bradykinin, and thus the BR1 signaling [105]. Moreover, these authors reported that LPS-mediated inflammation downregulated ACE2 bioavailability by a NFkB-involved mechanism. Of note, AngII induces NFkB expression through AT1R [45].

Aging does not only affect the KKK system through the ACE/ACE2 balance, but also directly altering the pharmacology of BR1 and BR2. It has been observed that although the serum levels of kinins increase with age, the responsiveness of target cells is limited or altered [106]. In this respect, bradykinin-induced vasorelaxation is actually affected by the BR1/BR2 ratio in the vasculature [107]. In older subjects, the density of BR2 is reduced whereas that of pro-inflammatory BR1 seems to be elevated, thereby changing the balance towards a vasoconstrictor response [108] that could result more deleterious in the context of SARS-CoV-2 infection. Most tellingly, both aging and kinins up-regulate the expression of pro-inflammatory COX-2 in several tissues [109–111].

In summary, the aging-related re-adaptation of the RAAS, KKK and COX-2 pathways may put older people in a new equilibrium situation much more sensitive to minor fluctuations and with a limited margin of response, rendering them more susceptible to inflammatory processes. These mechanisms and the ACE isoform D are summarized in Fig. 3c as crucial factors increasing COVID-19 severity.

## Conclusions: towards endotypification of COVID-19

**Fig. 4:**
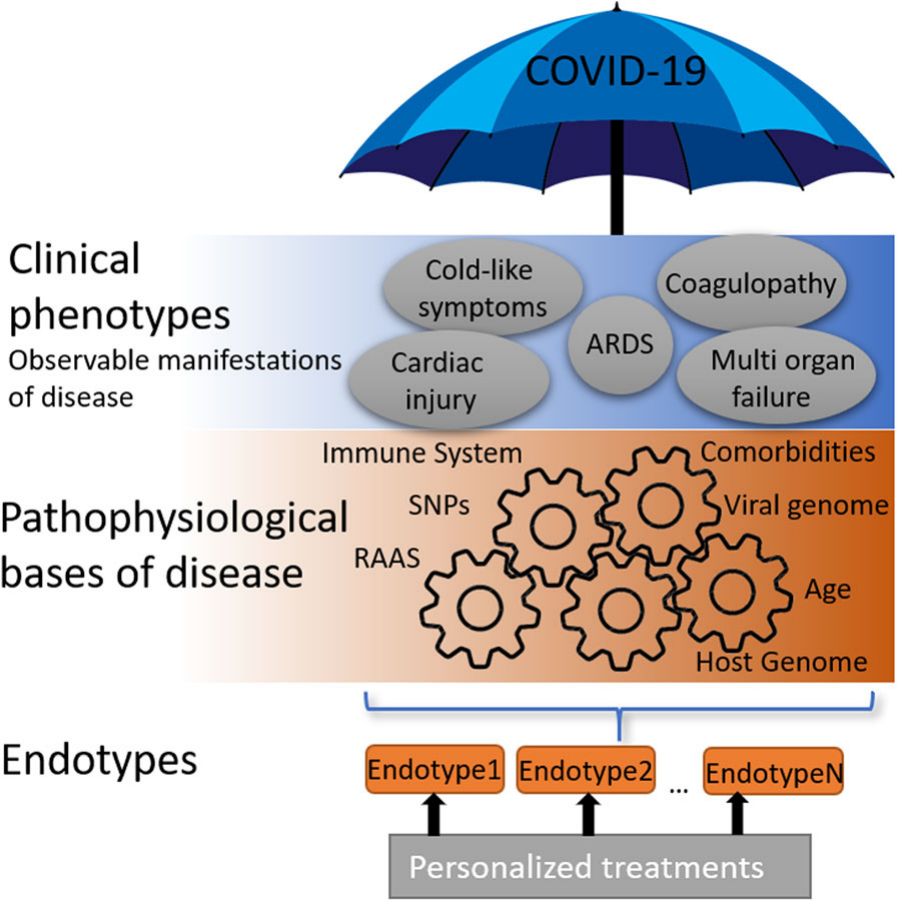
COVID-19, with its associated degrees of severity, is likely an umbrella term encompassing multiple pathophysiological bases of disease. Endotype discovery should dramatically accelerate our understanding and treatment of disease(s), enable further discovery of pathophysiological mechanisms and lead to identification of specific groups of patients that may benefit from personalized treatments

We are experiencing the first global pandemic since the dawn of precision medicine: an approach that leaves out a “one-drug-fits-all” model, in favor of customization of healthcare. In this context, identifying different endotypes —subtypes of a condition with different underlying pathophysiological mechanisms— should become central for clinical research because it helps to rationalize experimental results and enhances reproducibility: heterogeneous groups of patients consisting of varying unidentified endotypes are prone to obfuscate statistical analysis of clinical trials for potential vaccine candidates and therapeutic treatments and hinder the identification of different factors that modulate disease severity, among others. Endotype discovery has been particularly successful in the treatment of other respiratory illnesses, such as asthma [112] and bronchiolitis [113, 114] usually combining trajectory analysis of meaningful variables along time, cytokine profiles and multi-omics analysis. We speculate there is mounting evidence showing that COVID-19, with its associated degrees of severity and heterogeneous symptomatology, is actually an umbrella term that may include several endotypes (Fig. 4). The available data about age, gender, genotypes, polymorphisms, comorbidities and symptoms of disease points to the existence of different endotypes, with a probable central role of the RAAS involved in severe cases. Most SARS-CoV-2 cases are asymptomatic, with reports ranging between 50 to 70% of total cases [103]. The remaining occurrences are further split between mild, presenting cold-like symptoms, and severe cases. Considering the current lack of absolute numbers regarding total infections, it is likely that the percentages of severe cases are overestimated, and these may be further subdivided between i) those with other underlying factors, ii) others that are aggravated by comorbidities, and iii) those that are specifically affected by imbalance of the RAAS throughout the infection process. We hypothesize that there is a strong possibility that this particular subset of individuals are thrown off-balance by SARS-CoV-2 infection, constituting a distinctive endotype. For these patients, personalized treatments should address critical open questions such as how to manage ACE inhibitors [115] that are used in clinical practice for treating hypertension and other cardiovascular diseases: Although ACE inhibitors do not interfere directly with ACE2 activity [17], discrepancies exist regarding their effects on ACE2 expression levels in different tissues [116–118] raising the question of whether these drugs would be harmful for COVID-19 patients. Despite this, current consensus is to continue treatment until conclusive data emerge [119–121].

A constellation of factors may underlie the particular susceptibility of a RAAS-imbalanced endotype. Single nucleotide polymorphism (SNP) present in ACE2 can be classified as harmful or protective, depending on their effect on the binding affinity of the S/ACE2 complex [122], rendering them as possible factor underlying severity across different populations [123]. Aging may predispose to an exacerbated inflammatory response by downregulation of ACE2 and upregulation of COX-2, and gender, genotypes, SNPs and hypertension may play similar roles. Differences in the prevalence of comorbidities among sex -males are more likely to present comorbidities than females- may also partially explain the increased incidence (44 to 76% in males vs 24 to 56% in females) and mortality (55 to 64% in males vs 36 to 45% in females) observed in COVID-19 male patients [124]. These factors are expected to intersect at the regulation of ACE2: low expression levels render individuals particularly vulnerable to SARS-CoV-2, that in turn further downregulates ACE2 levels through shedding, critically affecting RAAS, bradykinin and COX-2 function. In particular, COX-2 may directly be affected by the interaction with ***N*** and ***S*** proteins. This is expected to onset proinflammatory mechanisms that are likely to establish a positive feedback with the ongoing viral infection, thus resulting in pneumonia and the observed cytokine storm, prothrombotic activity, and many of the severe symptoms detected in COVID-19. Although lower levels of ACE2 expression may seem protective as it would hinder viral entry, they appear to play a key role in the onset of severe symptomatology.

Other identified or proposed key factors that must be considered to identify different underlying endotypes include antibody-dependent enhancement [125], the role of previous infections with other coronaviruses, immunological profiles and genetic variations [9, 11–13]. A critical discussion of risk factors, comorbidities, pathophysiological basis of disease and their translational applications within the appropriate theoretical framework is prone to enable better understanding of the molecular basis of disease and, therefore, the design of successful strategies for personalized treatments.

## Data Availability

Because this is a review article, no individual data in any form is included inside the manuscript.

## Abbreviations

ACE: Angiotensin-converting enzyme
ACE2: Angiotensin-converting enzyme II
ADAM17: ADAM metallopeptidase domain 17
Ang-(1–7): Angiotensin-(1–7)
AngI: Angiotensin 1
AngII: Angiotensin 2
ARDS: Acute respiratory distress syndrome
AT1R: AngII receptor type 1
COX-2: Cyclooxygenase-2
HCoV: Human coronaviruses
KKK: Kallikrein-kininogen-kinin system
N: Viral nucleoprotein protein
NFKB: Nuclear factor kappa-light-chain-enhancer of activated B cells
RAAS: Renin-angiotensin-aldosterone system
RBD: Receptor binding protein
S: Viral spike protein
TMPRSS2: Transmembrane Serine Protease 2
